# Data-Driven Decision Support Tool Co-Development with a Primary Health Care Practice Based Learning Network

**DOI:** 10.12688/f1000research.145700.2

**Published:** 2024-12-19

**Authors:** Jacqueline Kueper, Jennifer Rayner, Sara Bhatti, Kelly Angevaare, Sandra Fitzpatrick, Paulino Lucamba, Eric Sutherland, Daniel Lizotte

**Affiliations:** 1 Department of Epidemiology and Biostatistics, Western University, London, Ontario, Canada; 2 Department of Computer Science, Western University, London, Ontario, Canada; 3 Centre for Studies in Family Medicine, Western University, London, Ontario, Canada; 4 Department of Research and Evaluation, The Alliance for Healthier Communities, Toronto, Ontario, Canada; 5 Health Information Systems Department, Compass Community Health, Hamilton, Ontario, Canada; 6 South Riverdale Community Health Centre, Toronto, Ontario, Canada; 7 Toronto Diabetes Care Connect, Toronto, Ontario, Canada; 8 Chatham-Kent Community Health Centres, Chatham, Ontario, Canada; 9 Expert in Digitalisation of Health Systems, Paris, France

**Keywords:** Primary Health Care, Decision Support Tool, Artificial Intelligence, Machine Learning, Diabetes, Mental Health, Descriptive Epidemiology, Electronic Health Records

## Abstract

**Background:**

The Alliance for Healthier Communities is a learning health system that supports Community Health Centres (CHCs) across Ontario, Canada to provide team-based primary health care to people who otherwise experience barriers to care. This case study describes the ongoing process and lessons learned from the first Alliance for Healthier Communities’ Practice Based Learning Network (PBLN) data-driven decision support tool co-development project.

**Methods:**

We employ an iterative approach to problem identification and methods development for the decision support tool, moving between discussion sessions and case studies with CHC electronic health record (EHR) data. We summarize our work to date in terms of six stages: population-level descriptive-exploratory study, PBLN team engagement, decision support tool problem selection, sandbox case study 1: individual-level risk predictions, sandbox case study 2: population-level planning predictions, project recap and next steps decision.

**Results:**

The population-level study provided an initial point of engagement to consider how clients are (not) represented in EHR data and to inform problem selection and methodological decisions thereafter. We identified three initial meaningful types of decision support, with target application areas: risk prediction/screening, triaging specialized program referrals, and identifying care access needs. Based on feasibility and expected impact, we started with the goal to support earlier identification of mental health decline after diabetes diagnosis. As discussions deepened around clinical use cases associated with example prediction task set ups, the target problem evolved towards supporting the upstream task of organizational planning and advocacy for adequate mental health care service capacity to meet incoming needs.

**Conclusions:**

This case study contributes towards a tool to support diabetes and mental health care, as well as lays groundwork for future CHC EHR-based decision support tool initiatives. We share lessons learned and reflections from our process that other primary health care organizations may use to inform their own co-development initiatives while we continue to work on advancing the population-level capacity planning model.

## Introduction

### Background

Increasing amounts of everyday data coupled with advancements in technology and artificial intelligence (AI) and its major subfield of machine learning are transforming healthcare. ^1^^–^^5^ Primary health care settings have received less attention than other sectors, and there is a need for increased engagement of end-users in development of AI-enabled decision support tools. ^6^^,^^7^ This case study describes the process and lessons learned thus far in co-developing a decision support tool with and for a primary health care organization in Ontario, Canada.

### Setting

The Alliance for Healthier Communities (Alliance) supports team-based primary health care through 72 Community Health Centres (CHCs) across Ontario, Canada to people who otherwise experience barriers to care. In 2000, all CHCs moved towards a common electronic health record (EHR; Telus Practice Solutions, version code 5.23.100) with standardized data requirements; each client’s EHR has structured fields for sociodemographic characteristics (e.g., sex, gender, education) and dynamic care encounter tables (e.g., ENCODE-FM *v7.2024* or ICD *-10* codes to indicate diagnoses and procedures) that capture information from all providers in their care team. All CHCs follow a standardized opt-out consent process for the use of de-identified data in research that reports aggregate results, which the analyses in this report fall under. De-identified data with consent for research use were stored on a secure server, with access through encrypted channels granted only as needed to members of the research team. The project was approved by the Western University Health Science Research Ethics Board in March 2018 (ID 111353).

### Report structure

**Figure 1.**
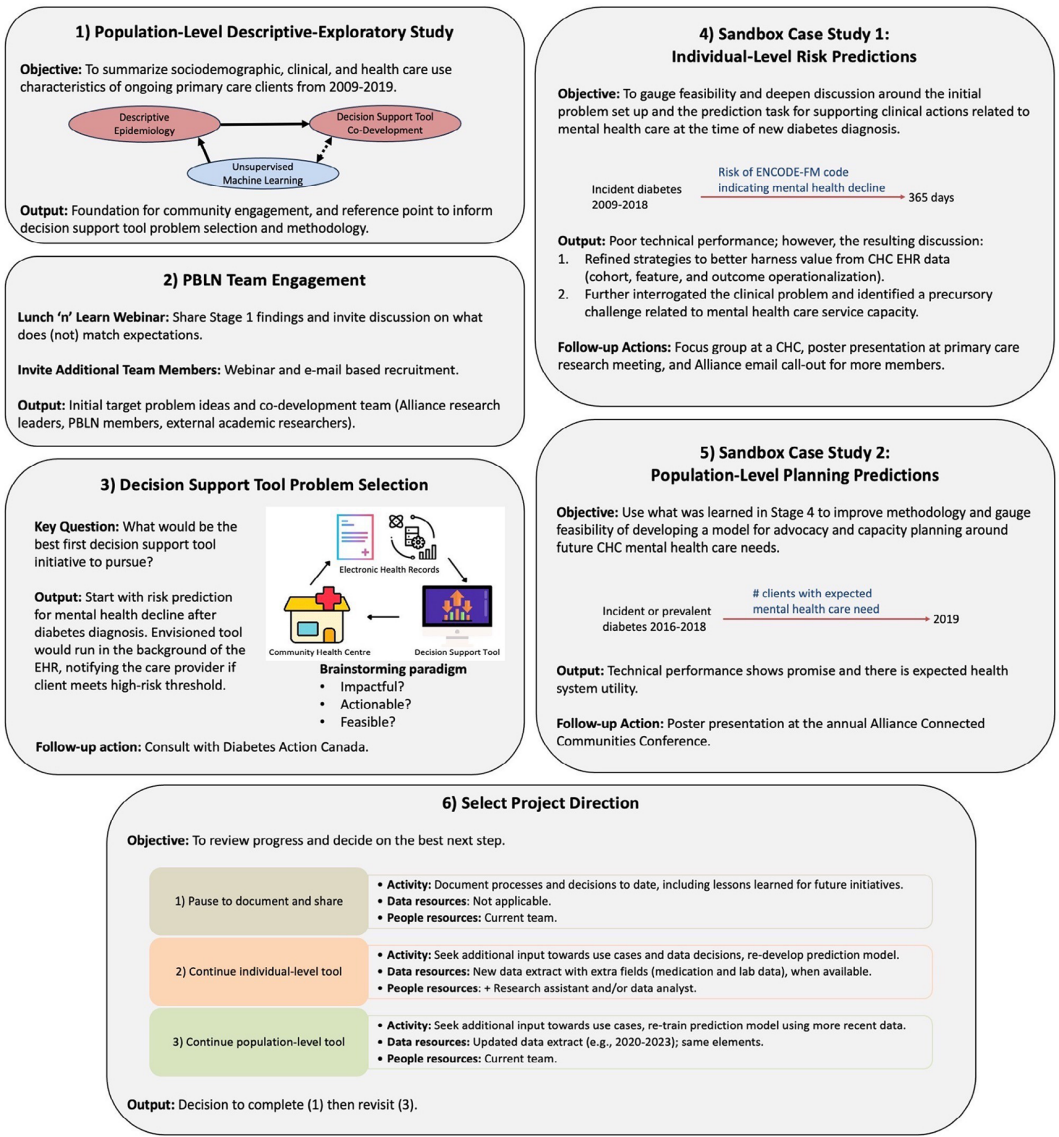
Case study overview. First six stages of decision support tool co-development project. Legend: Alliance = Alliance for Healthier Communities, CHC = Community Health Centre, EHR = Electronic Health Record.

Figure 1 summarizes our work completed thus far, with additional details for the six major stages of work presented below in terms of goals and associated activities. The discussion section summarizes overarching lessons learned and reflections.

## Stages of work

The Alliance has been heavily involved in setting quality EHR standards (including the collection and use of sociodemographic and race-based data) and in research studies, but this was their first EHR data-driven decision support tool co-development project. Thus, we started with a strategy to better understand how client characteristics and care patterns are represented in EHR data, and to motivate and facilitate initial brainstorming around meaningful challenges amenable to support with EHR-based data analysis tools.

### Stage 1: Population-Level Descriptive-Exploratory Study

Goals

1. To summarize sociodemographic, clinical, and health care use characteristics of ongoing primary care clients served through CHCs across Ontario from 2009 through 2019.
2. To serve as a foundation for community engagement and to inform decision support tool problem selection and methodological decisions.

Activities

**Conduct study:** This study (published elsewhere ^8^) provided an overview of population health and care patterns by applying to EHR data both methods from traditional descriptive epidemiology (e.g., period prevalence of chronic conditions) and from unsupervised machine learning to explore more complex patterns (e.g., non-negative matrix factorization to examine care provider teams). Findings were shared with the Alliance community through a Lunch ‘n’ Learn Webinar in October 2022, and were revisited throughout the decision support tool project to inform problem selection and methodological decisions.

### Stage 2: PBLN Team Engagement

Goals

1. To engage the broader Alliance community in critical thinking and discussion around secondary uses of EHR data.
2. To invite participation in the decision support tool co-development project.

Activities

**Population data assessment:** The Lunch ‘n’ Learn included an introduction to machine learning and decision support tools in addition to a summary of Stage 1 study findings. Embedded throughout the latter portion were polls and discussion points asking whether findings were consistent with expectations. Areas of inconsistency motivate further exploration to discern if the mismatch is an artifact due to data quality issues or methodological decisions, or if the mismatch is clinically relevant. Areas of potential concern (e.g., accurately high prevalence estimate) could be a good target for a decision support tool.

**PBLN team formation:** The Lunch ‘n’ Learn was the launching point of the decision support tool project, with additional invitations to participate distributed through the general Alliance community e-mail list and the recently formed PBLN member e-mail list. The resulting core team of people involved in remaining stages of work, described by general roles:

• Alliance research leaders (Director of Research, Research and Evaluation Project Lead): Provide input towards project process and all content/decisions, as well as coordinate engagement with the PBLN.
• PBLN members (care providers, clinical staff, IS staff ): Engage in critical discussion around the target problem and broader decision support tool initiatives, provide input towards methodological decisions, and review analysis findings.
• External researchers (professor, postdoctoral associate): Facilitate discussion sessions, lead analyses, and summarize findings.

### Stage 3: Decision Support Tool Problem Selection

Goal

1. To identify meaningful challenges within CHCs that are amenable to support with data-driven decision support tools.

Activities

**PBLN meeting:** In October 2022, the first PBLN meeting was held. We briefly reviewed descriptive epidemiology findings and discussed whether any outstanding questions needed to be answered to guide future steps. We then discussed data-driven decision support tools and brainstormed potential directions to pursue in the CHC context.

**Discussion synthesis:** Ideas were summarized into three “types” of decision support—risk prediction or screening, triaging specialized program entry, and identifying care access needs—with target conditions or application areas for each type ( Table 1).

**Table 1.** Candidate project directions by type of decision support with envisioned use and target application areas.

Type of decision support	Example decision support tool use	Priority application areas
**Risk prediction or screening**	Passively run in background of EHR system to predict outcomes, with the option to alert when a client reaches a high-risk threshold	Diabetes and mental health
**Triaging specialized program entry**	Predict who may benefit most from any given program or care option, used to support decisions when there is limited capacity in the program or in a client's care regime	Case conferencing or social prescribing
**Identifying care access needs**	Identify outstanding care needs among the population of interest, for staff to initiate proactive engagement of clients to support appointment completion, scheduling, or referral	Missing continuity of care or provider type(s) to add to a client’s care team

**PBLN meeting:** In December 2022, we reviewed the three candidate project directions and while all (and more!) were seen as potentially valuable we decided to focus on one while exploring how to best conduct this type of project: risk prediction for mental health decline after diabetes diagnosis. Rationale included i) *expected impact* (e.g., high prevalence of diabetes with known mental health comorbidities, coupled with the challenge of needing to choose between many possible care options upon a new diabetes diagnosis); ii) *actionable* (e.g., all CHCs provide mental health care resources that could help prevent mental health decline for people living with diabetes); and iii) *feasibility* (e.g., relevant care captured in the EHR, and heavy focus on risk prediction in machine learning methods advancements).

**Follow-up action:** We connected with Diabetes Action Canada to learn more about related work and potential collaborators. Given the early nature and CHC-specific focus of our project, we proceeded with risk prediction model development using an existing 11-year retrospective extract of CHC EHR data, with the intention to consider expansion or tighter external collaboration after more internal feasibility and impact assessments.

### Stage 4: Sandbox Case Study 1 – Individual-level risk predictions

Goal

1. To gauge feasibility and deepen discussion around developing a decision support tool that predicts early mental health decline within a year of incident diabetes indication.

Activities

**Preliminary analysis:** Candidate cohort summary characteristics, and an outline of potential predictor and outcome definitions and data sources.

**PBLN meeting:** In February 2023, we met to review the preliminary analysis and made initial decisions on the cohort and how to operationalize the outcome and predictors. This was done alongside further problem refinement and discussing clinical actions that could accompany a high-risk indication (Examples: brief educational discussion, noting down mental health to discuss in future appointments, referral to a specialized mental health provider, or referral to a CHC group program focused on mental health and/or diabetes).

**Model development:** The eligible cohort included 1,250 adult ongoing primary care clients receiving care at an East Toronto region CHC, who had at least one diabetes ICD-10 code ^9^ in 2011-2018, at least one year of follow-up care, and no mental health care or decline indication in the two years prior to their incident diabetes indication. The outcome prevalence representing mental health decline after new diabetes diagnosis was 8.4%. Five candidate models ranging in complexity from simple linear (sklearn.linear_model. LogisticRegression v0.24.2) to complex machine learning techniques ( CatBoost v0.26.1) were trained and compared using a five-fold nested cross validation procedure. Hyperparameters were selected on the inner loop using a grid search for the highest Area Under the Receiver Operating Characteristic Curve (AUROC).

**Model performance:** Table 2 presents summary performance metrics, with additional methods and results details available upon request.

**Table 2.** Numbers represent average performance across the five outer test folds.

	Logistic regression	Lasso logistic regression	CatBoost - features	CatBoost - encodes	Hybrid model
**Stage 4: Individual-level risk prediction model performance**					
**AUROC**	0.60	0.65	0.68	0.67	0.63
**AUPRC**	0.14	0.15	0.15	0.15	0.15
**Stage 5: Population-level planning prediction model performance**					
**AUROC**	0.78	0.78	0.89	N/A	N/A
**AUPRC**	0.53	0.53	0.73	N/A	N/A

Legend: AUROC = Area Under Receiver Operating Characteristics Curve (measure of discrimination, the ability to assign a higher risk score to someone randomly selected with the outcome than someone randomly selected without the outcome; higher is better), AUPRC = Area Under the Precision Recall Curve (measure of the tradeoff between precision and recall performance; higher is better), CatBoost-Features includes sociodemographic and preprocessed clinical features (e.g., count of chronic conditions), CatBoost-Encodes includes sociodemographic features and clinical information as more granular ENCODE-FM codes, Hybrid model = Hybrid feature and similarity based model ^16^ with sociodemographic features and kernel-based similarity of ENCODE-FM codes.

**PBLN meeting:** In April 2023, we met to review and revise the initial model development analyses. While we decided the predictive performance of the sandbox model was not clinically useful, the associated discussion surfaced ideas around how to better harness information from the data and around problem refinement. Example insights included broadening eligibility from incident to prevalent cases of diabetes, modifying how to identify active diabetes care, and surfacing the need to engage more people to better understand how different provider types code mental health care in the EHR (e.g., do ENCODE-FM codes for “feeling anxious” vs “anxiety” reliably distinguish symptoms vs. diagnosis or is this more indicative of different provider type scopes). In terms of features, medication and lab value data are expected to increase accuracy of individual-level predictions; these data are planned for integration with the BIRT system (supporting data access), but not yet readily available.

**Follow-up actions:** We sought further input on the case study and suggested next steps in three ways: a PBLN member lead a focus group at their CHC, a poster presentation was given at a primary care research gathering, and an email call out for further input was circulated through the Alliance email listserv.

**Problem refinement:** A strong discussion theme was that while all CHCs provide mental health services, these are already at or near capacity and implementation of the decision support tool may increase demand past a point that could be maintained. This highlighted potential value of instead developing a system-level decision support tool to address the upstream problem of how to plan or advocate for adequate capacity within CHCs to address future mental health care service needs.

### Stage 5: Sandbox Case Study 2 – Population-Level Planning Predictions

Goal

1. To use what was learned in Stage 4 to develop a sandbox model to predict the number of ongoing primary care clients with prevalent diabetes indications who will have mental health care needs in the upcoming year.

Activities

**Model development:** Example methodology changes from the Stage 4 case study were loosening eligibility criteria to include clients already receiving mental health care, broadening the outcome to include additional ENOCDE-FM codes (categories: emotional symptoms, symptoms involving appearance, suicidal ideation, affective disorder, and anxiety), and changing the missingness strategy for sociodemographic variables (collapsed not asked with no answer). We performed a similar five-fold nested CV procedure but restricted to feature-based models due to discussions (supported by case study 1 results) that preprocessed counts of chronic conditions should be more informative than granular codes.

**Figure 2.**
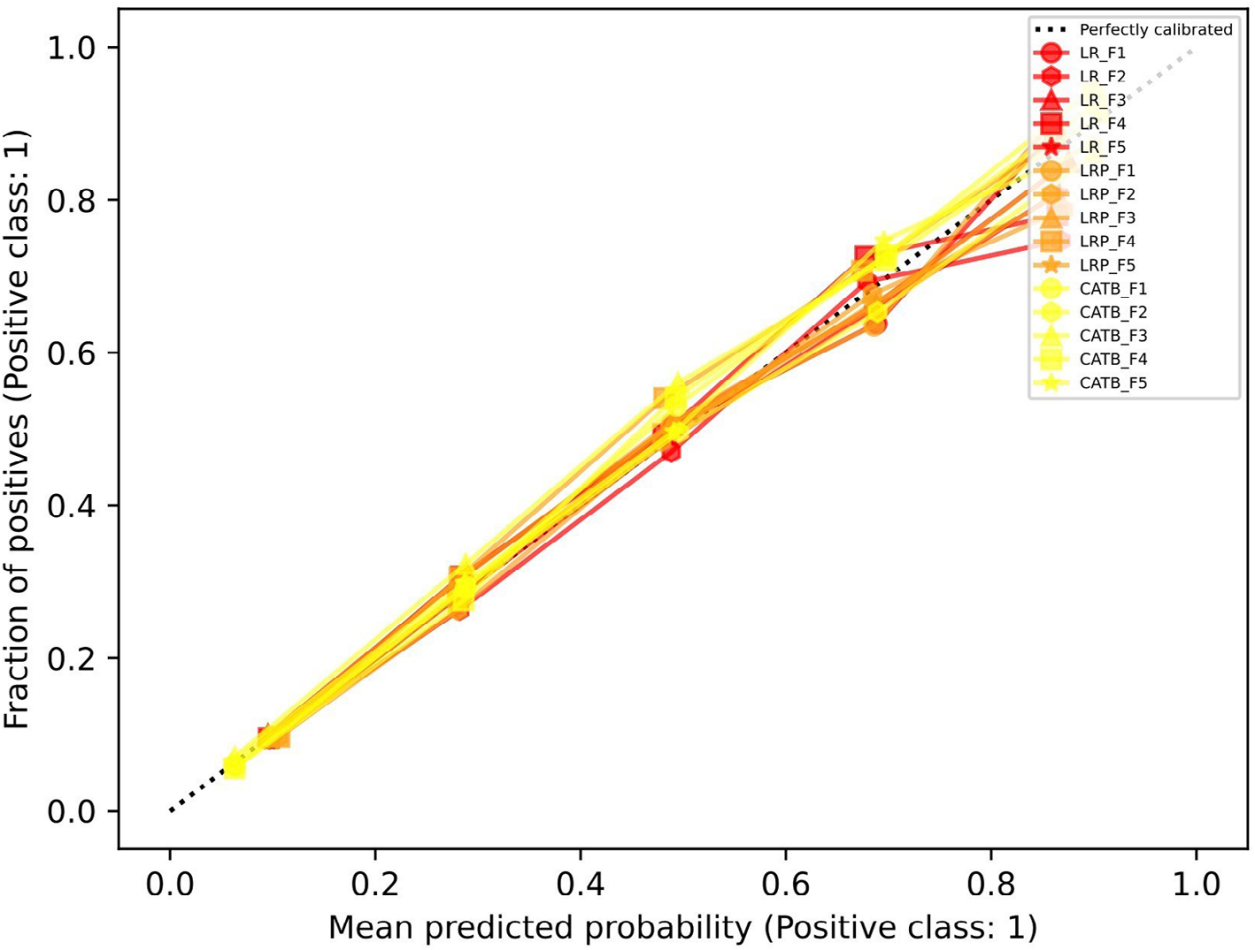
Outer fold calibration performance for population-level prediction results. Legend: LR = Logistic Regression, LRP = LR with L1 penalty, CATB = CatBoost, F# = Fold number in cross validation procedure.

**Model performance:** Of the 20,329 eligible clients with prevalent diabetes in 2016-2018, 22.2% had a mental health care outcome recorded in 2019. We used a naïve 0.5 probability cut-off for the best performing model ( CatBoost v0.26.1) across all outer test folds to demonstrate the type of information that could be made available from this type of model to support capacity-planning decisions or advocacy. Overall model accuracy was 86%; CHC-specific accuracy calculated as the average accuracy across the five outer folds stratified by clients belonging to each CHC ranged from 64% to 97%, plus one CHC where there were no predicted or actual outcome cases (100% accuracy). The CHC-specific proportion of clients with diabetes predicted to have mental health care needs ranged from 46% (vs. actual 48%) to 1% (vs. actual 13%). Calibration performance is in Figure 2, and AUROC and AUPRC metrics are in Table 2.

**Follow-up actions:** We presented on all stages of the project at the annual Alliance Conference, ^10^ and discussed the second case study at a PBLN meeting in July 2023. The sandbox model predictive performance showed promise and discussions further supported the idea that this type of advocacy and capacity planning tool would be a beneficial precursor to the individual-level tool in terms of actionability and associated clinical or system utility.

### Stage 6: Project Recap and Next Steps Decision

Goal

1. To review progress thus far and select which project direction to pursue next.

Activities

**Figure 3.**
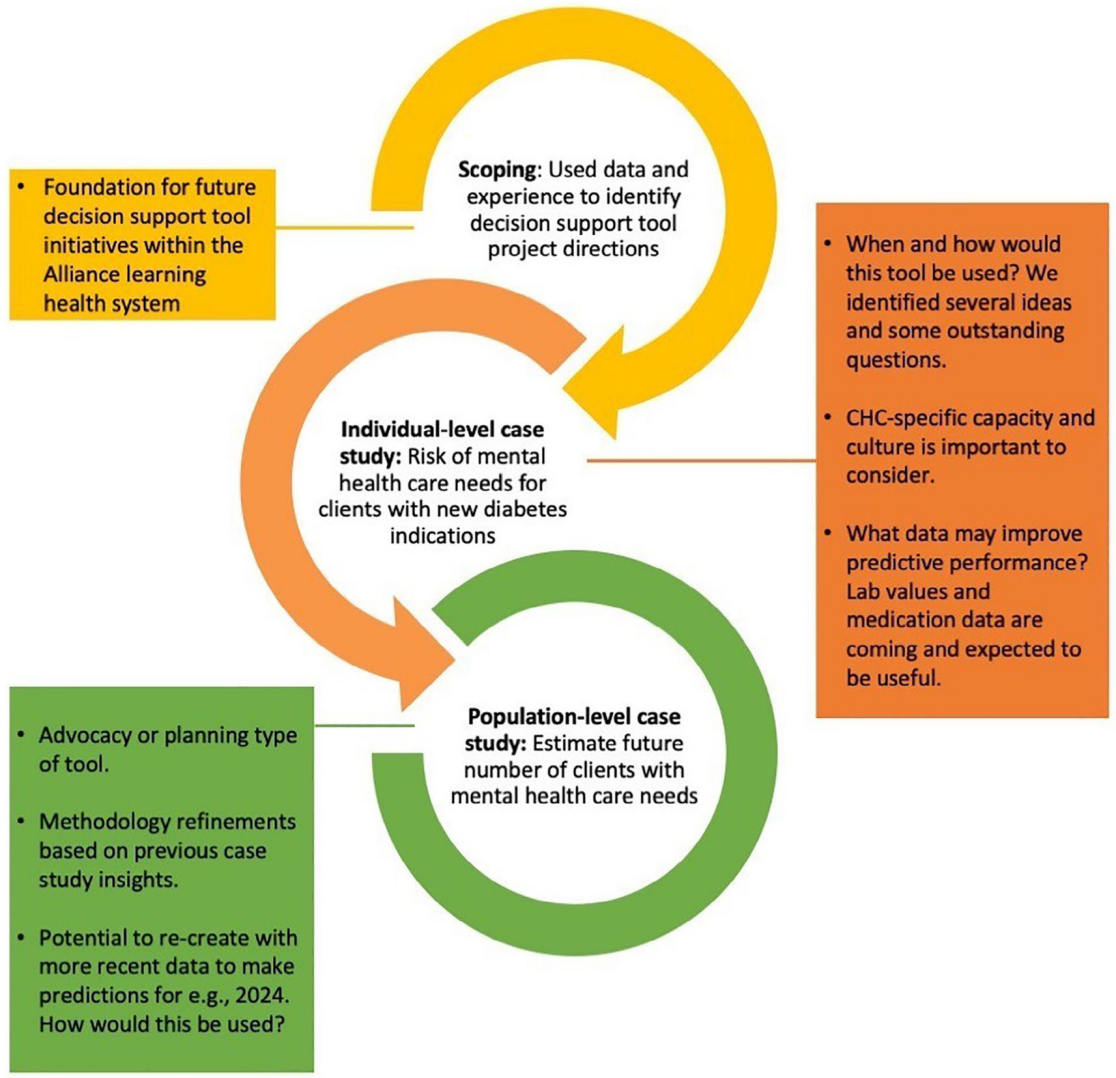
Summary of major progress and insights thus far. Legend: Alliance = Alliance for Healthier Communities, CHC = Community Health Centre.

**PBLN meeting:** In addition to discussing case study 2 results, **t**he July 2023 PBLN meeting included a review of project progress summarized into three points ( Figure 3)—project scoping and problem identification, case study 1, and case study 2—alongside potential next steps with expected resource needs.

**Next steps:** Given the novelty of this project within the Alliance and the broader field of machine learning-enabled technologies for primary health care, the technical and process related progress made thus far towards a fully functional tool to support diabetes and mental health care additionally includes lessons that would benefit future projects of different focus. Therefore, we decided to pause to document our processes before revisiting the population-level planning tool direction.

## Discussion

### Summary

Our decision support tool co-development approach with and for the Alliance started with a large-scale epidemiological study to learn about health and care patterns of the population of interest and about how clients are represented in CHC EHR data. We identified a priority problem of supporting proactive mental health care for clients with diabetes, and iterated between sandbox case studies and discussion sessions to further understand the problem, how a data-driven decision support tool could provide support, and the feasibility of achieving a high-quality technical solution given readily available EHR data.

### Reflections and lessons learned

• **Epidemiology as a foundation for innovation:** We used epidemiology as a launching point to supplement clinical and organizational expertise in brainstorming meaningful problem selection. Example benefits: 1) provided an overview of population health and care patterns (e.g., diabetes and mental health prevalence estimates informed expected impact assessment), 2) supported understanding around data quality and how clients are represented in aggregate EHR data (e.g., reliably recorded data elements as part of feasibility assessment), and 3) informed methodological decisions during model development (e.g., the first year of care at a CHC has a distinct profile wherein incidence and prevalence are hard to parse). Furthermore, this rigorous population-level overview ^8^ can inform multiple initiatives outside the current project scope, and provides a baseline for longer-term evaluation or monitoring of the impact of EHR-based tools over time and with continued use.
• **Importance of an interdisciplinary team:** Our team included clinical, research, technical, IS, and organizational leadership expertise. This allowed for seeding discussions with examples, critically assessing meaningful or realistic use of candidate tool ideas in practice and in the context of the organization—starting with direct envisioned uses (e.g., brainstorm clinical actions in response to X type of alert) and extending into how to follow through on those actions amidst competing demands at an individual client care level and at a system capacity and culture level. For example, moving from the problem of how to support individual level mental health and diabetes care to address the more pressing challenge of system-level advocacy for adequate resources to be able to follow-through on best care options that would otherwise result from the original tool idea. Including different perspectives further helped to scope the project in terms of what data are available and when, identify the best methodology to meet clinical goals, connect with internal and external collaborators, and support feasibility and continuity from a system-capacity perspective.
• **Sandbox case studies supported deeper discussion sessions:** The impact of the sandbox case studies can be likened to how editing a manuscript often matures or builds on ideas relative to when writing the first draft. They were particularly valuable given the novelty of this type of project within the Alliance and the limited amount of research literature on co-development of machine learning-enabled decision support tools for primary health care settings. Even when predictive performance of a sandbox model was poor, the tangible example pushed discussions further than hypothetical scenarios or thought experiments could. Discussing why specific technical decisions did or did not line up with realistic or impactful clinical scenarios improved problem conceptualization, and the resulting methodological decisions can be applied to future projects. Of note, development of the “real” model intended for deployment will use a new data extract with more recent data.
• **Problem scoping around data availability and quality:** The Alliance has put significant effort towards their data platform and quality as part of their learning health system journey, including standardization and integration of data from all care team members. ^12^ This rich EHR data capturing information about multiple domains of health increased the feasibility of our project; however, the target problem and methodology also needs to consider what data are available and to what extent for the population and intended implementation sites of interest. For example, completeness of sociodemographic characteristics varies across CHCs and certain data types (e.g., medications, lab tests) flagged as potentially valuable for our first sandbox case study were scheduled to become available about a year out, which influenced our problem refinement.
• **Multiple engagement strategies are needed:** The initial Lunch ‘n’ Learn session coupled with email list invitations was effective for forming the core project team, which included approximately five to twenty members in attendance at meetings. In seeking further input towards the project, a PBLN member led focus group at their CHC was more effective than research poster presentations or further e-mail-based recruitment. The focus group resulted in highly-relevant and actionable feedback.
• **Working towards a broader decision support tool initiative:** As experience is gained, tool development will become more efficient and effective. While there are hundreds of potential targets for decision support tools within the broad scope of primary health care, it will not be sustainable or beneficial to continue to create or implement these independently. A fragmented approach to tool integration poses risks such as exacerbating alert fatigue or disrupting instead of augmenting team-based, whole-person care. Rather, our broader vision is to support more seamless integration of multiple types of tools to benefit people (e.g., better health outcomes), providers (e.g., improved workflow and decisions support), and communities (e.g., sufficient resources to meet demands). An additional consideration for expansion will be when to maintain a tool consistently across all CHCs versus adapt it to the local CHC context and available data.

This project more generally furthers the Alliance’s learning health system work at three levels: data analysis capacity, stakeholder engagement, and process refinement. ^11^^–^^15^ First, demonstrating the use of machine learning to make data more meaningful through large-scale descriptive and real-time, clinically relevant predictive insights that will ultimately improve care delivery. Second, providing new avenues for clinician and provider engagement in data-driven learning initiatives; future work will additionally be able to engage the newly formed Client and Community Research Partners Program. Third, by providing a baseline process for tool development that future projects can learn from and build upon (i.e., what stages of work to keep, modify, or replace). Each future decision support tool initiative will provide additional opportunities for bidirectional learning whereby data are harnessed through AI and machine learning to tackle a specific clinical problem and improve care delivery, while simultaneously learning how to improve and adapt the processes to achieve that clinical goal for different types of decision support and application areas.

## Conclusions

This case study describes the ongoing process and lessons learned thus far in the first EHR-based decision support tool co-development project with and for CHCs in Ontario, Canada. Since sharing our initial report, we have continued to refine and validate the population level prediction model with more recent data, and held additional focus groups with staff, providers, and clients that informed an updated outcome definition and cohort eligibility criteria to better align with capacity planning goals. Our current focus is still on diabetes and mental health care, with a vision of extending into a larger and longer-term decision support tool initiative that would integrate multiple types of tools with the CHC EHR system. Our processes and reflections may further inform or motivate other primary health care organizations at a similar stage of learning how to best harness value from EHR data.

### Ethics and consent

Consent for use of de-identified data for research purposes, whereby results are only made available in aggregate, was collected through a standardized opt-out procedure followed by the Community Health Centres where care was provided. Consent is collected via a written form, which is then scanned into the EMR. The need for additional consent specific to the research in this report was waived by the Western University Health Science Research Ethics board (ID 111353).

## Data Availability

This project includes the following underlying data: 11 years of electronic health records for adult clients who had at least one encounter at an Ontario Community Health Centre in 2009-2019. The data extract includes a client characteristics table (e.g., date of birth, level of education completed) and dimension and fact tables including details about care encounters over time (e.g., diagnostic codes, providers involved, referrals). Due to their sensitive nature, the data are securely stored and not shareable for ethical reasons, as outlined by the Western University Research Ethics Board (ID11353). Inquiries about access for future research studies should be directed to the Department of Research and Evaluation, Alliance for Healthier Communities ( lhs@allianceon.org).
