# Supplementary material for: Data-Driven Decision Support Tool Co-Development with a Primary Health Care Practice Based Learning Network
Source: F1000Res. 2024 Dec 19;13:336. Originally published 2024 Apr 23. [Version 2] doi: 10.12688/f1000research.145700.2 (PMC11809626; doi:10.12688/f1000research.145700.2)
Supplement: Peer review history

# Reviewer report: Reviewer response for version 2

**DOI:** 10.5256/f1000research.176019.r350738

**Published:** 2025-01-09

**Authors:** Nicole M White

**Affiliations:** 1 Queensland University of Technology, Brisbane, Queensland, Australia

Thank you for sending the revised version. Authors have addressed all feedback in their response and subsequent revisions.

Is the case presented with sufficient detail to be useful for teaching or other practitioners?

Partly

Is the work clearly and accurately presented and does it cite the current literature?

Yes

If applicable, is the statistical analysis and its interpretation appropriate?

Partly

Are all the source data underlying the results available to ensure full reproducibility?

No

Are the conclusions drawn adequately supported by the results?

Yes

Is the background of the case’s history and progression described in sufficient detail?

Yes

Reviewer Expertise:

Biostatistics, health services research

I confirm that I have read this submission and believe that I have an appropriate level of expertise to confirm that it is of an acceptable scientific standard.

# Reviewer report: Reviewer response for version 1

**DOI:** 10.5256/f1000research.159682.r303167

**Published:** 2024-08-16

**Authors:** Nicole M White

**Affiliations:** 1 Queensland University of Technology, Brisbane, Queensland, Australia

This study reports on a multi-stage process for developing and refining a clinical decision-support tool using EHR primary care data. Six main stages are described, starting with an epidemiological study to motivate stakeholder discussions to identify areas that may benefit from decision support and tool development. Two case studies are presented, involving the use of different classification algorithms to predict mental health decline and population-level service demand, respectively.

 The framework is well designed and described throughout the manuscript. Strengths of the framework are stakeholder involvement in both development and refinement stages, and the use of data and preliminary results to motivate these discussions.

 Given the emphasis on end-user engagement, it would be beneficial to include some high-level data on engagement levels at different stages. This additional information could be described in the Stages of work section and/or Reflections and lessons learned section. For example, the authors provide information on which engagement strategies worked better than others – how many participants were recruited from the ‘best’ engagement strategy and were numbers above/below/in line with expectations?

 Whilst the model development itself is not the focus of this study, these sections would benefit from more information in the relevant stages of work (4, 5) and the Discussion. I noted that the metrics chosen to summarize model performance were not consistent between case studies. Making these metrics more consistent would strengthen the paper and reporting transparency. For model development in stage 4, some information on outcome prevalence would help to gauge the suitability of the stated sample size and its impact on model performance. Model developed information for Stage 5 would also benefit from clarifying the difference between the five-fold CV procedure and CHC-specific results. I interpreted the latter as being the results of internal-external model validation.

 In the Discussion, the study alludes to future work once more data are available. Whilst having more recent data may contribute to building a better model, data quality is equally important. It would be useful to include some comments on data quality in both case studies for context, and whether quality was a barrier. This is particularly pertinent to EHR data streams known to be of variable quality.

 As a minor comment, the terms ‘AI’ and ‘ML’ are used interchangeably throughout the manuscript. Whilst these disciplines are overlapping and complementary, using these terms interchangeably can make it difficult for the reader to understand what specific methods were used.

Is the case presented with sufficient detail to be useful for teaching or other practitioners?

Partly

Is the work clearly and accurately presented and does it cite the current literature?

Yes

If applicable, is the statistical analysis and its interpretation appropriate?

Partly

Are all the source data underlying the results available to ensure full reproducibility?

No

Are the conclusions drawn adequately supported by the results?

Yes

Is the background of the case’s history and progression described in sufficient detail?

Yes

Reviewer Expertise:

Biostatistics, health services research

I confirm that I have read this submission and believe that I have an appropriate level of expertise to confirm that it is of an acceptable scientific standard, however I have significant reservations, as outlined above.

# Response

**Published:** 2024-12-14

**Authors:** Jacqueline Kueper

**Affiliations:** Epidemiology and Biostatistics, Western University, London, Ontario, Canada

Dear Dr. Nicole M White,

 Thank you for your interest and review of our article. We appreciate your suggestions for improvement and have addressed them as summarized below, resulting in a more robust and transparent article. 

 Regarding end-user engagement levels, unfortunately we did not collect any formal attendance records or statistics. We have however added to the ‘reflections and lessons learned’ section an approximate size of the team and more details about why the focus groups were considered successful.

 Regarding model development, we agree that additional details would be helpful and have broadened Table 2 to include performance metrics (AUROC, AUPRC) from both Stage 4 and Stage 5 so that they can be compared. We also followed your suggestion to add the outcome prevalence for the Stage 4 cohort (8.4%), and further clarified how the CHC-specific results were calculated (stratification by groups of clients belonging to each CHC, from the same data as for the overall metrics).

 Regarding data quality, we agree that data quality is as if not more important than recency. Working within limited space, we expanded the Reflections section on data availability to include both availability and quality, included some high level reflections from our experience that the relatively high quality of data from CHCs increased the feasibility of our study, and referenced a study that did a more in-depth assessment of data from CHCs as part of the organizations journey to being a learning health system.

 Finally, thank you for flagging the confusion between AI and machine learning throughout. We added to the opening sentence clarification that we refer to machine learning as a major subfield of AI and then updated remaining text about the stages and lessons learned to include machine learning as that is the family of techniques we used. One exception is we left AI in the final sentence of the lessons learned section as we wanted to capture the broader scope of methods that may be used in a learning health system and future projects, but added in machine learning to make it explicit we also mean to include the techniques described earlier.

# Reviewer report: Reviewer response for version 1

**DOI:** 10.5256/f1000research.159682.r283425

**Published:** 2024-05-31

**Authors:** Ana De Marchi, Ericles Bellei

**Affiliations:** 1 Institute of Health; Institute of Technology, University of Passo Fundo (Ringgold ID: 28129), Passo Fundo, State of Rio Grande do Sul, Brazil; 2 Institute of Health, University of Passo Fundo (Ringgold ID: 28129), Passo Fundo, State of Rio Grande do Sul, Brazil

The study demonstrates the development and application of a data-driven decision support tool within a primary health care setting, employing a methodologically sound iterative process that is both reflective and inclusive of stakeholder engagement. The use of both traditional epidemiological methods and advanced machine learning techniques enriches the analytical depth and application potential of the decision support tool, offering a comprehensive approach to addressing primary health care challenges. The documentation of the co-development process provides a valuable blueprint for similar future initiatives in health informatics.

 This study's dedication to including a wide range of stakeholders throughout the development process is one of its most noteworthy strengths. It guarantees that the tool is not only theoretically sound but also practically relevant and easy for clinical practitioners to utilize. This strategy increases the likelihood of acceptance and successful use in practical contexts, which is in line with the study's goals of enhancing primary healthcare delivery through cutting-edge technological innovations.

 However, as the authors mention that the project is ongoing, I suggest for the conclusion section (and by extension, the abstract) to provide more detail about the current status of the initiatives and planned future studies. This would offer a clearer understanding of the project’s trajectory and ensure that the study’s documentation keeps pace with its development, thereby reinforcing the relevance and timeliness of the research. This adjustment would help maintain a clear narrative through to the study's completion and beyond, as it progresses from methodology formulation to practical application.

 As a reviewer, I would like to point out that given the case study format of this research, it does not delve into detailed descriptions of the Population-Level Planning Predictions models. These models are likely to be more comprehensively detailed in a separate, dedicated study. Therefore, a deeper evaluation of these models is not feasible within the scope of this case study.

Is the case presented with sufficient detail to be useful for teaching or other practitioners?

Yes

Is the work clearly and accurately presented and does it cite the current literature?

Yes

If applicable, is the statistical analysis and its interpretation appropriate?

Not applicable

Are all the source data underlying the results available to ensure full reproducibility?

Partly

Are the conclusions drawn adequately supported by the results?

Yes

Is the background of the case’s history and progression described in sufficient detail?

Yes

Reviewer Expertise:

Digital health; Participatory design; Primary care

We confirm that we have read this submission and believe that we have an appropriate level of expertise to confirm that it is of an acceptable scientific standard.

# Response

**Published:** 2024-12-14

**Authors:** Jacqueline Kueper

**Affiliations:** Epidemiology and Biostatistics, Western University, London, Ontario, Canada

Dear Dr. Ana De Marchi and Dr. Ericles Bellei,

 Thank you for your interest and review of our work. It is helpful to see what stands out as strengths from your perspective, and we appreciate the suggestion to include additional details about the ongoing project. Accordingly, in a revised version we added a sentence to the conclusion about how we are refining and validating the population-level model with updated data, and that we have held additional focus groups within the health system to get further insights. These focus groups lead to an updated outcome definition that is better tailored to mental health capacity planning as well as a broadening of the cohort eligibility from ongoing primary care clients to also including diabetes education center clients who may or may not also be primary care clients.
